# Functional Hydrophilic Membrane for Oil–Water Separation Based on Modified Bio-Based Chitosan–Gelatin

**DOI:** 10.3390/polym13071176

**Published:** 2021-04-06

**Authors:** Siti Zarina Zakuwan, Ishak Ahmad, Nurfaizah Abu Tahrim, Faizal Mohamed

**Affiliations:** 1 Department of Chemical Sciences, Faculty of Science and Technology, Universiti Kebangsaan Malaysia, Bangi Selangor 43600, Malaysia; nfaizah@ukm.edu.my; 2 Department of Applied Physics, Faculty of Science & Technology, Universiti Kebangsaan Malaysia, Bangi Selangor 43600, Malaysia; faizalm@ukm.edu.my

**Keywords:** hydrophilic membrane, oil–water separation, gelatin, chitosan, biopolymer membrane, microstructure, filtration

## Abstract

In this study, we fabricated a modified biomaterial based on chitosan and gelatin, which is an intrinsic hydrophilic membrane for oil–water separation to clean water contamination by oil. Modification of the membrane with a non-toxic natural crosslinker, genipin, significantly enhanced the stability of the biopolymer membrane in a water-based medium towards an eco-friendly environment. The effects of various compositions of genipin-crosslinked chitosan–gelatin membrane on the rheological properties, thermal stability, and morphological structure of the membrane were investigated using a dynamic rotational rheometer, thermogravimetry analysis, and chemical composition by attenuated total reflectance spectroscopy (ATR). Modified chitosan–gelatin membrane showed completely miscible blends, as determined by field-emission scanning electron microscopy, differential scanning calorimetry, and ATR. Morphological results showed membrane with establish microstructure to further experiment as filtration product. The membranes were successfully tested for their oil–water separation efficiencies. The membrane proved to be selective and effective in separating water from an oil–water mixture. The optimum results achieved a stable microporous structure of the membrane (microfiltration) and a separation efficiency of above 98%. The membrane showed a high permeation flux, generated as high as 698 and 420 L m^−2^ h^−1^ for cooking and crude oils, respectively. Owing to its outstanding recyclability and anti-fouling performance, the membrane can be washed away easily, ensuring the reusability of the prepared membrane.

## 1. Introduction

Major oil pollution does not come from oil spills from large tankers, but from other sources, such as industrial and domestic drainages. The widespread use of oil in human life and industries increases the percentage of oil pollution and has eventually become a global challenge for the ecosystem. This is due to the lack of supervision, e.g., discharging oily wastewater into water resources without treatment. Oil has several applications, such as its use in vehicles (petrochemicals), food, plastics, lubricants, and the textile and paint industries. Oily wastewater or waste oil can have varying viscosity, volatility, and toxicity, which determine how detrimental it is for human health and the environment [1]. Different types of oils can be difficult to remediate depending on the oil spill intensity and the location. A certain oil spill in water leaves a residue of up to one-third of the spilled volume after a few days with possible long-term contamination in intertidal regions [2].

Over the last few decades, membrane separation has been primarily used in wastewater treatment for separating oil and water. It requires only simple equipment and has high efficiency and low energy requirements. The method used for treating oil spill contamination is carefully selected to optimize the operational cost and minimize the environmental effects [3,4]. Several synthetic separation materials have been used because of their low cost and effectiveness in removing oil from wastewater or oil spills. However, synthetic polymer membranes are easily fouled, causing a decreased flux and separation efficiency [1]. The hydrophobic membrane polymer resulted in their contamination especially during the oil–water separation filtration process, leading to the decrease of water flow efficiency [5]. The fabrication of membranes with high performance that are eco-friendly and created using sustainable, bio-based material has attracted significant interest. These membranes can also offer low energy consumption, low membrane fouling issues, and no degradation due to heating. Bio-based natural resources are considered promising substitutes or complements to their synthetic counterparts, which are water-permeating and hydrophilic-underwater surfaces, to increase the membrane efficiency and life and prevent oil fouling effects [6]. Polysaccharide chitosan and protein gelatin are well-known materials for making such bio-membranes. The membrane production technology is environmentally friendly and uses fewer or no chemicals compared to synthetic membrane fabrication, which makes it more sustainable and beneficial to the economy. Therefore, in recent times, membrane technology based on biopolymers has proven to be an attractive option for wastewater-treatment processes [7].

Chitosan is a non-toxic cationic polymer consisting of β-(1,4)-D-glucosamine and N-acetyl-D-glucosamine, and it is the second most abundant natural biopolymer after cellulose [8]. Furthermore, chitosan contains one –NH_2_ group and two –OH groups in each glycosidic unit. Chitosan-based materials have also been suggested as potentially eco-friendly coagulants and flocculants for water and wastewater treatment because of their natural biological characteristics and biodegradability [9]. The advantage of chitosan which has unique properties such as disinfectants, due to higher antibacterial activity shows the ability of chitosan to combine with other biopolymers [9,10]. Gelatin contains amino acids, and it is an inexpensive biopolymer with the ability to form a gel. It acts as a texturizing, thickening, and water-binding material with good stability due to its biocompatibility with other biopolymers. Gelatin molecules assemble into a network of triple helices, forming a thermo-reversible, viscoelastic gel upon cooling [11]. The main limitation of gelatin in producing membrane foam is its rapid dissolution in aqueous media. Owing to its antimicrobial and adhesion properties, chitosan provides a unique combination that has excellent potential to act as a biopolymer membrane in membrane separation. Both gelatin and chitosan exhibit good biocompatibility, biodegradability, and commercial availability. However, these two materials are fragile and have poor mechanical properties and an unstable structure, which limit their ability to provide sufficient mechanical strength in an aqueous environment for effective oil–water separation [12,13].

Therefore, materials made from chitosan and gelatin are required to overcome limitations such as poor resistance against aqueous media, which can deteriorate its structure. Moreover, the materials are required to maintain membrane structure integrity. Previous studies have shown that to maintain the structural integrity of the gelatin–chitosan materials in long-term applications, gelatin and chitosan should be crosslinked [8,14]. Hence, there is a demand for a natural crosslinker such as genipin, which is a potentially good crosslinking agent owing to its non-toxicity compared to chemical crosslinkers such as aldehyde compounds. Furthermore, the chemical crosslinkers such as aldehyde compounds have high toxicity and produce poisonous products upon reaction with chitosan and gelatin, thereby limiting their use in biomaterial production and application. Genipin has biocompatible crosslinked products with cytotoxicity 10,000 times lower than glutaraldehyde. These findings encouraged us to utilize genipin as a green modifier to produce novel functional membrane applications, a natural product with low cytotoxicity that focuses on environmental problems [8,15,16]. In addition, the water-soluble crosslinker 1-ethyl-3-(-3-dimethylaminopropyl)carbodiimide (EDC) is non-toxic; however, achieving uniformity and a low degree of crosslinking are problems that limit the application of this crosslinker [17]. The main limitation of physical crosslinking, such as ultraviolet (UV)- and γ-irradiation and dehydrothermal (DHT) treatment, is that these methods depend on time and technique for protein crosslinking and are less efficient for obtaining high crosslinking materials. The limitation can be overcome by combining the physical crosslinking methods with appropriate chemical methods [18,19,20]. In this study, the main constituents are bio-based; genipin is a natural product that was obtained from geniposide, isolated from the fruits of Genipa Americana and Gardenia Jasminoides Ellis [21,22], whereas the dark blue pigments were obtained by the reaction of genipin with the primary amine. We produced a series of chitosan–gelatin crosslinked membranes with different compositions and investigated their physicochemical properties. Moreover, we evaluated their potential applications in oil–water separation, through the process of separation using a membrane, with the process of separation that uses only the force of gravity. The gravity separation process, which separates two immiscible oil–water feed mixtures according to their densities, is simple and conventional.

The aim of this study was to produce an eco-friendly filter membrane with antifouling properties to clean water that contained high amounts of greasy/oily substances (used engine oil) and fats (waste cooking oil). Oily substances must be removed from aqueous systems because of the increasingly serious environmental pollution caused by their abundant discharge in industrial and domestic drainages. Reusability is a key feature of this membrane; it is possible to remove material that causes it to clog by cleaning the surface and removing oily contamination.

## 2. Materials and Methods

### 2.1. Reagents and Materials

Pharmaceutical-grade gelatin was purchased from Halagel (M) Sdn. Bhd. (Kuala Lumpur, Malaysia). Low-molecular-weight chitosan was purchased from Sigma-Aldrich (St. Louis, MO, USA). Glacial acetic acid (99.5%) and glycerol (99.5%) were purchased from SYSTERM-chemAR (Malaysia) and were used as plasticizers to prepare the bio-membrane. Genipin was purchased from Challenge Bioproducts Co., Ltd. (Taichung, Taiwan). Petroleum crude (CRUDE OIL) C0280 was purchased from Progressive Scientific Sdn. Bhd. (Malaysia).

### 2.2. Preparation of Membrane

Gelatin was dissolved in distilled water and heated to 50 °C with moderate stirring to form a homogeneous gel. Chitosan was dissolved in a 1% (*v*/*v*) acetic acid solution until completely dissolved. Various compositions—with chitosan:gelatin blending ratios of 1:3, 1:5, and 1:7—were prepared separately. Further, known amounts of the gelatin and chitosan solutions were poured together and stirred for 2 h at 70–90 °C. Then, 1% (*w*/*w*) of genipin was added to the mixture and dispersed with magnetic stirring. Each mixture was kept at 50 °C under moderate stirring to gelatinize, and the color of the whole solution changed from transparent to blue during crosslinking. The resultant hydrogel was left at approximately 23–26 °C for 4–5 days to allow complete gel formation. Further, it was lyophilized at a temperature of −45 ± 5 °C, using a vacuum dryer, to obtain porous membrane foam.

### 2.3. Attenuated Total Reflectance Infrared Spectroscopy (ATR-IR)

Infrared spectra of the membrane samples were recorded with an ATR mode at ambient temperature in the range of 4000–700 cm^–1^ on a model 2000 Perkin Elmer instrument (Hopkinton, MA, USA) equipped with a diamond ATR crystal. For characterization, samples were cut into specimens of dimensions 1 cm × 1 cm × 0.2 cm and placed on the ATR plate to determine the possible interactions between the functional groups in the crosslinked gelatin–chitosan blend.

### 2.4. Mechanical Testing

Mechanical performance of a membrane sample was evaluated by measuring the tensile strength and modulus, using a universal testing machine (Instron model 5566, Norwood, MA, USA) at room temperature. During these tests, 5 mm/min cross speed, 40 mm initial grip distance, and 50 N cell load were used. The membrane foam thicknesses were determined at five points, using a digital caliper (Mitotuyo, Kuala Lumpur, Malaysia, ±0.02 mm). For each sample, the average value of seven repetitions was obtained.

### 2.5. Rheological Analysis

The rheological properties of the polymers were measured by using an Anton Paar Physica MRC 301 dynamic rotational rheometer (Anton Paar GmbH, Graz, Austria) operating in the strain-controlled mode. Viscosity data were obtained by using a shear rate gradient of 0.05–500 s^–1^ with a dynamic mode frequency sweep (strain control). The moduli and complex viscosities were recorded.

### 2.6. Morphological Analysis

Morphological analysis of the cross-section fracture membrane was also performed by using field emission scanning electron microscopy (FESEM) (Philips XL-3, North Billerica, MA, USA). The membrane was cut into small pieces, and all the samples were coated with gold before observation.

### 2.7. Thermogravimetric Analysis (TGA)

The thermal weight loss and degradation behavior of the crosslinked samples were studied using a thermogravimetric analyzer (Mettler Toledo, TGA/SDTA 851-E, Columbus, OH, USA) under a nitrogen atmosphere from 25 to 1000 °C at a heating rate of 10 °C min^–1^.

### 2.8. Differential Scanning Calorimetry (DSC)

To evaluate thermal stability of the membrane foam sample, calorimetry measurements were performed by using a differential scanning calorimeter (Mettler Toledo-DSC 822e, Columbus, OH, USA). The samples were heated to 200 °C, at a rate of 10 °C min^–1^, under a nitrogen atmosphere.

### 2.9. Swelling Degrees

The swelling degrees of the membranes were determined by evaluating their ability to swell in distilled water at 25 °C for 24 h. The samples were dried before immersion in distilled water. Swelling is an important property of biodegradable materials to determine the percentage of water absorption. Herein, swelling studies were performed by immersing the scaffolds in distilled water. The dry weight of the sample was determined before immersion. The sample was immersed in distilled water and taken out after a predetermined time of 24 h. Further, surface adsorbed water was removed by a filter paper and the wet samples weights were recorded. The ratio of swelling was determined by using the following equation:

(1) $$\mathrm{Swelling}\;\mathrm{ratio}=\;\frac{\left(w_{2}-w_{1}\right)}{w_{1}}\times 100$$

where *w_2_* and *w_1_* are the hydrated weight and dried weights of the membrane, respectively.

### 2.10. Membrane Testing

The membrane foam was tested initially at the lab scale, and the test was conducted under gravity. A mixture of oil and water was poured into a transparent glass apparatus for monitoring. For each sample, the tests were repeated three times and an average value was obtained. Further, the permeate flux of a membrane sample was calculated by using the following equation:

(2) $$\mathrm{Flux}=\frac{V}{At}$$

where *V* is the volume of water that permeated across the membrane, *A* is the area of the membrane (m^2^), and *t* is the time testing. Flux in Lm^−2^ h^−1^, *V* is the volume of permeate collected at time t for each test, and 150 mL was poured into the filter.

The laboratory samples of oil-in-water were prepared by mixing 20% (*w*/*w*) of oil (used engine oil, waste cooking oil, and light crude oil) and 80% (*w*/*w*) of water phase solution. The oil/water mixture underwent bath sonication for five minutes to form an emulsion.

## 3. Results and Discussion

### 3.1. ATR-IR

**Figure 1:**
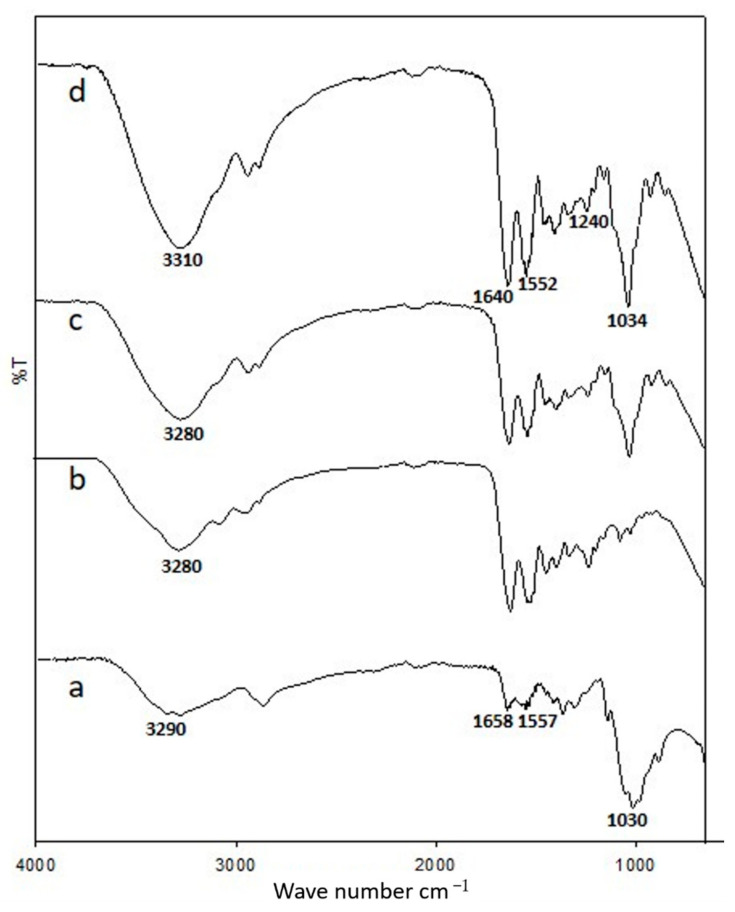
Attenuated total reflectance (ATR) spectra of (**a**) chitosan membrane, (**b**) gelatin membrane, (**c**) 1:7 chitosan–gelatin membrane (without crosslinking), and (**d**) 1:7 genipin-crosslinked chitosan–gelatin membrane.

Figure 1 shows the Fourier Transform Infrared spectra of the chitosan membrane, gelatin membrane, and non-crosslinked and genipin-crosslinked chitosan–gelatin membranes. The spectra show repeated functional groups present in the chitosan and gelatin samples with absorption peaks in the 3310–3280 cm^−1^ region, which are attributed to the partially overlapped stretching vibrations of hydroxyl and amine groups. Furthermore, the peak at 2940–2800 cm^−1^ is assigned to –CH_2_ and –CH_3_ groups. The strong absorption bands at 1640, 1552, and 1240 cm^−1^ in the spectrum of the gelatin sample correspond to the stretching vibrations of amide I due to the stretching vibrations of C=O, N–H bending of amide II, and N–H bending of amide III, respectively. As shown in Figure 1, the spectrum of the chitosan membrane exhibits a characteristic peak at 1658 cm^−1^, which corresponds to the carbonyl stretching mode of amide groups. Furthermore, the peak at 1557 cm^−1^ corresponds to the bending modes of primary and protonated amines [9]. The appearance of similar peaks in the spectrum of the chitosan–gelatin membrane due to the recognizable peaks of chitosan in the range of 1030–1034 cm^−1^ distinctly correspond to the C–N stretching vibrations and result from the crosslinking interaction between the genipin and chitosan [8]. Moreover, the peak in the range of 1640–1658 cm^−1^ is attributed to the formation of amide bonds, thereby confirming that the crosslinking occurred [21,23]. The presence of these peaks of chitosan and gelatin clearly indicates a successful combination of these two polymers and confirm ionic interaction between positively charged chitosan and negatively charged gelatin. The spectrum of chitosan–gelatin with genipin confirms that the characteristic absorption bands of the chitosan–gelatin blends remain unchanged with genipin crosslinking and are also present in the final product. Compared to the spectrum of the membrane without crosslinking, the spectrum of the genipin-crosslinked chitosan–gelatin membrane shows an increase in the intensity ratio of the amide and amine absorption bands, which confirms the reaction between chitosan–gelatin and genipin [24,25].

### 3.2. Mechanical Testing

**Figure 2:**
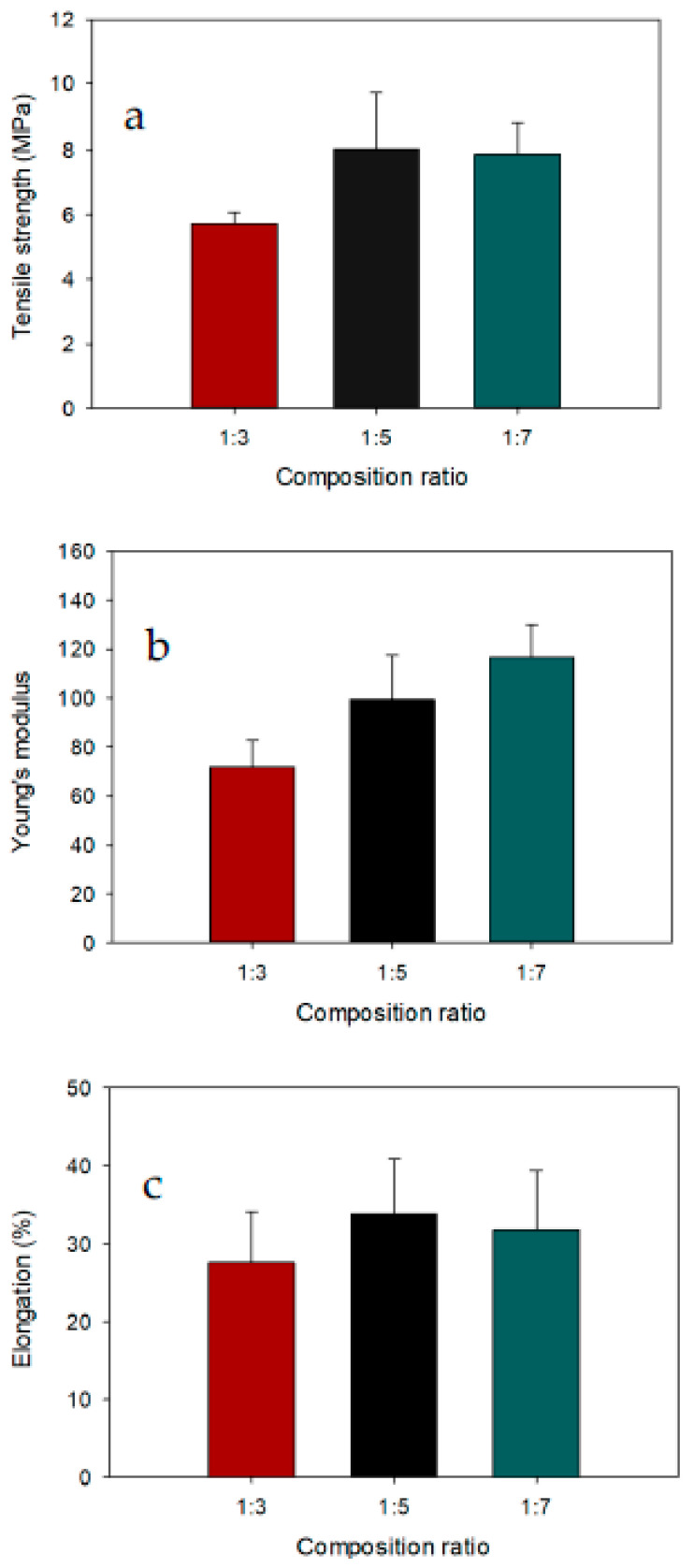
(**a**) Tensile strengths, (**b**) Young’s modulus, and (**c**) elongations at break (%) of modified chitosan–gelatin membrane foam with different chitosan:gelatin composition ratios.

Membrane foam strength is one of the important functional properties of bio-based membranes. The physical properties of the modified chitosan–gelatin membrane foam is attributable to the intermolecular and intramolecular hydrogen bonds. Low membrane foam strength limits its use in the oil/water separation process. These results clearly show that incorporating gelatin increased the tensile strength and Young’s modulus of the modified membrane compared to various composition ratios of chitosan to gelatin. The mechanical strength pattern of the modified chitosan–gelatin membrane as a function of gelatin content is shown in Figure 2. With increasing gelatin content, the strength of the genipin-crosslinked chitosan–gelatin membrane significantly increased, and the highest strength was observed for the chitosan:gelatin composition ratio of 1:7 compared to the membrane with the composition ratio 1:3 and 1:5. The membrane with the lowest gelatin content (1:3) had the lowest tensile strength at 5.71 MPa. The membrane with a chitosan:gelatin composition ratio of 1:5 exhibited a tensile strength of 8.05 MPa, which is higher than those of the other membranes (i.e., with composition ratios of 1:3 and 1:7). Higher gelatin content in the membrane with the composition ratio of 1:7 resulted in a tensile strength of 7.87 MPa. The slight decrease in tensile strength with no significant difference compared to 1:5 chitosan–gelatin membrane was most probably due to the porosity of the membrane, which negatively influenced the stress transfer during material testing.

The tensile modulus of the chitosan–gelatin membrane clearly showed that the stiffness of the samples increased with the increasing gelatin content. Among the membranes with different chitosan:gelatin composition ratios, the chitosan–gelatin membrane foam with a composition ratio of 1:7 had the highest modulus strength (116.7 MPa). Adding genipin to the membrane helped strengthen the membrane, and this was reflected in its structural stability, which provided sufficient mechanical strength in an aqueous environment for effective oil–water separation. Theoretically, genipin can change the protein structure through both intramolecular and intermolecular crosslinks, thereby leading to a balance of the mechanical properties. Therefore, the chitosan–gelatin membrane was strongly reinforced with genipin crosslinker, due to interactions between the chitosan–gelatin during the blending process, reflecting the interactions between the interfacial protein and continuous phase protein via both noncovalent and covalent linkages [26]. An increasing trend in the elongation of the membrane was observed with the addition of gelatin. The highest elongation of 33.8% occurred with a chitosan:gelatin composition ratio of 1:5. These observations for modified membranes can be explained by the molecular structure and reactivity of the crosslinking agent. Moreover, the molecular chain length of the crosslinking agent can affect the elasticity, brittleness, and crosslinking density of the structures [27].

### 3.3. Rheological Analysis

**Figure 3:**
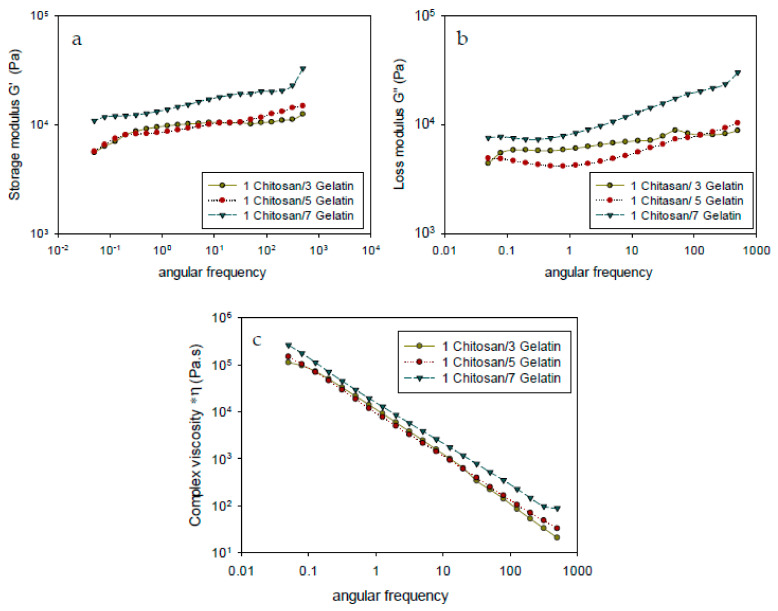
Rheological properties of modified chitosan–gelatin membrane foam with different composition ratios of chitosan:gelatin: (**a**) storage modulus (G’), (**b**) loss modulus (G”), and (**c**) complex viscosity (*η).

The results of rheological analysis of the chitosan–gelatin membrane with different compositions are shown in Figure 3, which shows the dynamic shear storage moduli and complex viscosity. In this study, the rheological properties defined the deformation and flow of matter to show the relationship between the processability behaviors, structural properties, and the three-dimensional crosslinked network structures of the materials formed with different compositions.

Figure 3a,b shows similar trends in the storage and loss modulus at different angular frequencies. All samples exhibited higher storage modulus (G’) than loss modulus (G”), which was mainly due to the stable crosslinked networks of the membrane. The increase in the gelatin content resulted in an increase in the storage modulus (G’), which corresponded to the enhanced crosslinked density of the membrane. The changes in G’ and G” corresponded to the energy changes occurring during the dynamic shear process and were strongly dependent on the interaction between the polymer interphases in the blend [28]. A higher gelatin content, as in the composition ratio of 1:7, resulted in a higher G’ value under shear flow due to the stronger covalent bonds within the gelatin network, which allowed more covalent binding predominantly to the amino acids of gelatin via chemical reaction with the crosslinker genipin [29]. In addition, in the chemical crosslinking reaction between chitosan and genipin, the self-associated network of chitosan led to a weak physical interaction with a permanent covalent network to form a stronger elastic membrane [30]. Hence, higher energy was required to overcome the strong interaction between the covalent bond of chitosan–gelatin with genipin due to the chain-expanding structure between the protein chains and crosslinked genipin chains in order to create covalent linkages and consequently increase entanglement.

Compared to the membrane with the composition ratio of 1:7, the membranes with the composition ratios of 1:3 and 1:5 exhibited a lower formation of new covalent bonds and the proteins and crosslinkers had a weaker association with each other as a result of the lower gelatin content. The highest elastic modulus was observed for the 1:7 membrane owing to the formation of the new networks through an interaction of amino acid with crosslinkers. For the membrane with increased gelatin, the increase in rheological properties, which resulted in a higher crosslinking density of the hydrogel, may be related to the strong intermolecular interactions of genipin with protein and contribute to forming a highly interconnected blending matrix. By increasing the gelatin content, the crosslinking with genipin increased due to the reaction of the amino groups of chitosan and gelatin with genipin in the blending solution, which led to the formation of macromolecular chains [14,26]. Thus, adding various amounts of gelatin to the chitosan systems increased both G’ and G” to different extents, thereby implying the reinforcement of the chitosan hydrogel network. The formation of a more rigid membrane led to a reduction in the swelling ratio (Section 3.7).

The complex viscosities (*η) of the modified chitosan–gelatin membrane is shown in Figure 3c. Complex viscosity (*η) is a measure of the total resistance to flow as a function of angular frequency (ω), which is the study of the change in flow characteristics of a substance under applied stress or force. The composition ratio affects the values of the complex viscosity (*η). These variations in the (*η) value is due to the varying gelatin content in the composition ratio. It can be seen that the complex viscosity of the membrane increases with the increasing gelatin content in the composition ratio of the membrane. Furthermore, Figure 3c shows that the complex viscosity decreases with increasing frequency. During the dynamic shear process at higher frequencies, the decreasing complex viscosity (*η) is related to the strong shear thinning behaviors of the blend in a solid state. It can be seen from Figure 3c that the membrane with the composition ratio of 1:7 has the highest *η, whereas no significant difference in the *η was observed for the membranes with the composition ratios of 1:3 and 1:5. Therefore, the increase in the value of *η with increasing gelatin content might be due to the formation of a network structure. A larger amount of gelatin content may cause a greater entanglement of the chains, and thus gradually increase the value of *η. High amino content in gelatin explains the increase in the *η value due to the strong intermolecular interactions of genipin with protein which new forming of covalent bonds between the amino acid side chain with genipin crosslinkers. Rheological behavior is sensitive to the molecular structure.

### 3.4. Morphological Analysis

**Figure 4:**
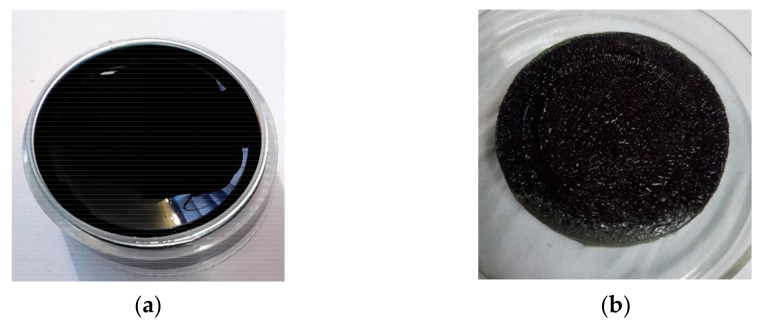
Photographic image of (**a**) hydrogel and (**b**) membrane foam of the chitosan–gelatin sample with the chitosan:gelatin composition ratio of 1:7.

The successful crosslinking of the chitosan–gelatin membrane with genipin was verified by the change in the color from transparent to dark blue. The photographic images of the hydrogel and membrane foam are shown in Figure 4. The hydrogel (Figure 4a) presents sample conditions after crosslinking and before the hydrogel was lyophilized. Then, the sample underwent a sublimation process to form a membrane. Previous research also reported that the formation of a deep blue pigment in the hydrogel during the process is a strong indication of a successful reaction between the primary amine groups of chitosan, gelatin, and genipin [15]. The hydrogel formed with sufficiently high interaction between chitosan, gelatin, and crosslinking genipin shows a clear dark blue color, is stable and stiffness, and can be used for further investigation and applications [31].

**Figure 5:**
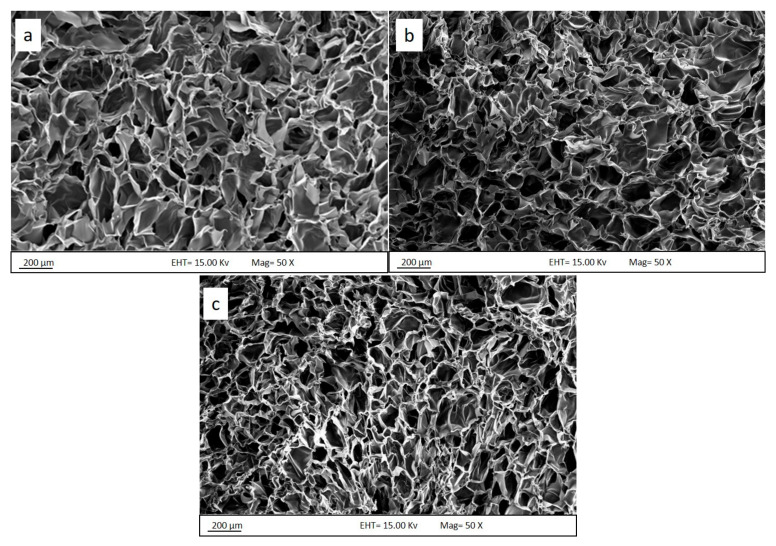
Cross-sectional FESEM images of the membrane foam in chitosan–gelatin composition ratios of (**a**) 1:3, (**b**) 1:5, and (**c**) 1:7.

The morphology of the porous membrane structure and the dimensions of the membrane play an important role in determining the properties of the membrane for the separation process. These characteristics were analyzed by using field emission scanning electron microscopy (FESEM). Figure 5 shows the cross-sectional FESEM images of the membrane with different composition ratios of chitosan:gelatin after lyophilization. The membranes exhibited a well-defined, highly interconnected porous network structure, as seen in the FESEM images. The cross-sections of the membranes indicate that the membranes were successfully produced with three-dimensional inner pores. The cross-sectional micrograph displays macro-pores and differences in geometry according to the composition ratio.

As seen in Figure 5, the pore dimensions, described by the FESEM micrographs, decrease with increasing gelatin content. The membrane structure showed significant differences and uniform structure formation. The structure with a higher gelatin content showed better interconnection between components. The pore size obtained from the cross-section had dimensions in the range of 100–200 μm. This was observed in membrane samples with different chitosan:gelatin composition ratios. Similar porosity diameters in the range of 150–200 μm were also reported for a chitosan–genipin sample [22], revealing a well-interconnected network with compact macro-domain porosity [15]. At the lowest gelatin content, the pore diameter of the chitosan–gelatin membrane was below 250 μm. The pore structure minimized gradually as the gelatin content increased. For example, the membrane with a chitosan:gelatin composition ratio of 1:7 exhibited relatively small pores with an average diameter of 100 μm and below. High porosity with stable structure is the most important physical indicator of a high permeation rate and stability at certain pressures that will allow the water to pass through the membrane foam. The permeation is not completely dependent on the special wettability of the produced membrane foam [32,33].

Furthermore, a previous study reported that the crosslinking reaction occurs on both chitosan and gelatin, resulting in the formation of an interacting polymeric network. The porous membrane of the materials appears like a sponge. This also reflects the strong interaction of chitosan and gelatin molecule chains reacting with each other by electrostatic interactions; hydrogen bonds are formed between gelatin and chitosan and are strengthened by genipin [34,35].

The chitosan–gelatin membrane sample with a higher gelatin content had a compact scaffold structure with decreased porosity, as shown in Figure 5c. This might be related to the higher content of gelatin, which tends to form membrane structures through crosslinking. The higher gelatin content in the membrane corresponded to more crosslinking points; therefore, the small structure pores linked together and produced more structural inner pores, inducing the formation of the smallest pores compared with the membranes with low gelatin content. In contrast, the membrane structure containing a lower content of gelatin formed large-dimension pores in the scaffold structure, which led to a slight increase in the pore size. As higher gelatin content in the composition conveyed higher mechanical strength, a membrane with a composition ratio of 1:7 also retained its original structure after the swelling test. The increase in the gelatin content enhanced the scaffold porosity structure.

### 3.5. Thermogravimetric Analysis

**Figure 6:**
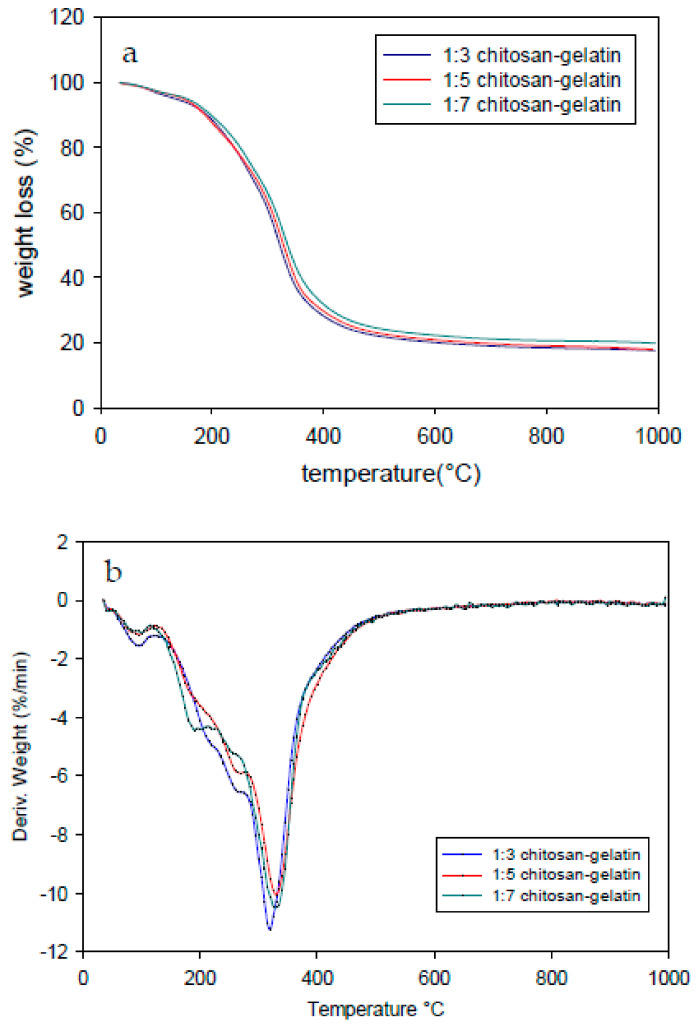
(**a**) Thermogravimetric analysis (TGA) and (**b**) derivative thermogravimetry (DTG) curves of the modified membrane foam in the chitosan:gelatin composition ratios of 1:3, 1:5, and 1:7.

Thermogravimetric analysis (TGA) was performed to verify the thermal stability of the membrane at the various composition ratios. Figure 6a, b shows the TGA and derivative thermogravimetry (DTG) of the samples, respectively. All the samples showed similar thermograms with slightly increasing degradation patterns with increasing gelatin content. Table 1 lists the maximum degradation rate temperatures (T_d_) of all the samples. The degradation trend was similar across composition ratios (1:3, 1:5, and 1:7). The initial thermal degradation that occurred between 50 and 150 °C for all the samples corresponded to the water evaporation in the materials. The second stage of weight loss appeared between 200 and 450 °C, corresponding to the thermal decomposition of the samples. The higher temperature of the thermal degradation stage, which exceeded 500 °C, initiated the real breakage of the membrane resulting from thermal destruction of the structure and the complete decomposition of the membrane sample. Most of the mass loss occurred in the second stage, as shown in the Figure 6. The higher composition of gelatin provided more functional groups that crosslinked and showed better thermal stability compared to those with lower gelatin content. A previous study showed the thermal stability of blended chitosan and gelatin in the range of 281–341 °C [24]. The decomposition process occurred in the range of 250–450 °C and was attributed to the degradation of the gelatin chain [36] and the crosslinked networks of amino groups in gelatin with genipin [26]. The second thermal degradation of the chitosan–genipin polymer can be attributed to the decomposition temperature in the range of 180–340 °C [27]. The degradation peaks shifted to higher temperatures with increasing gelatin content. The degradation of the modified chitosan–gelatin membrane did not occur with the two major separation processes during the thermal degradation process. This indicates no material separation between the gelatin and chitosan in the material produced. Blending chitosan and gelatin protonated the primary amino groups (NH^3+^) on chitosan, which combined with the carboxylate (COO−) ions of gelatin to form a polyelectrolyte network structure by a strong ionic bond. However, other intermolecular forces such as hydrogen bonds between the carboxyl (COOH), hydroxyl (OH), and amino (NH_2_) groups in chitosan and gelatin molecules will interact with the dipole [24].

A higher gelatin content resulted in a rigid network owing to the genipin crosslinking because of strong intermolecular interactions of genipin with the protein that contributed to the highly interconnected hydrogel bonding (interaction of chitosan–gelatin). The crosslinking modified the thermal degradation properties of the chitosan–gelatin membrane possibly as a result of the formation of ester and/or amide bonds between gelatin, chitosan, and genipin. Minimum residual masses of 17.60%, 19.91%, and 17.96% were obtained for the gelatin–chitosan membrane foam (1:3), (1:5), and (1:7) blends, respectively.

### 3.6. DSC

**Figure 7:**
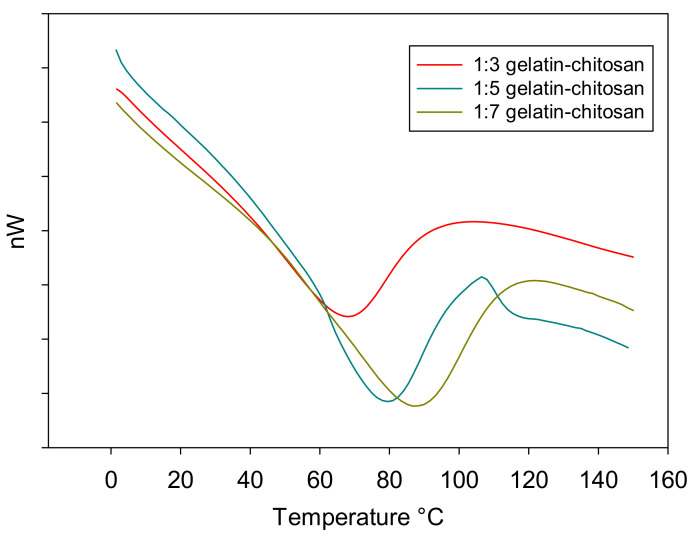
Differential scanning calorimetry (DSC) curve of the modified membrane foam at chitosan:gelatin composition ratios of 1:3, 1:5, and 1:7.

The thermal stability of the modified chitosan–gelatin membrane foam with various composition ratios was determined from the DSC data shown in Figure 7. The endothermic peak values are shown in Table 1. The peak values increased with the increasing thermal stability of the membrane, which is important for the application of chitosan–gelatin materials. The chitosan–gelatin thermogram presented an endothermic peak corresponding to the wide denaturation temperature (T_d_) range with a decomposition endothermic peak for chitosan:gelatin composition ratio of 1:3 at 67.95 °C. As shown in Figure 7, the peak values of the crosslinked membrane chitosan–gelatin shifted toward higher temperatures compared to the value of the membrane with a lower gelatin content. The thermograms show the DSC curve of the chitosan:gelatin composition ratios of 1:5 and 1:7 show the transition temperature in an enhanced transition temperatures at 79.28 °C and 87.30 °C, respectively. In addition, these studies on the thermal stability of gelatin modified by crosslinkers is of great interest because of its application in oil–water separation, where the membrane may experience sustained heat during their processing and preparation during the production process, or consumption during the application process. An increase in the denaturation temperature shows an increase in the number of crosslinks per molecule with an increasing gelatin content in the composition ratio. As reported previously, genipin-modified proteins had higher denaturation temperatures due to the significant increase in the transition temperature from 67 to 87 °C with the increase in the gelatin content, which can be attributed to the stronger protein–protein interaction during the blending of chitosan–gelatin. Therefore, genipin crosslinking indicates a higher thermal stability [37]. In addition, the higher denaturation temperature reflected a greater degree of crosslinking or stabilization according to the T_d_ was an indirect measurement of the degree of crosslinking because the higher the crosslinking degree of the material’s higher entanglement chain after crosslinking process [38].

### 3.7. Swelling Degrees

**Figure 8:**
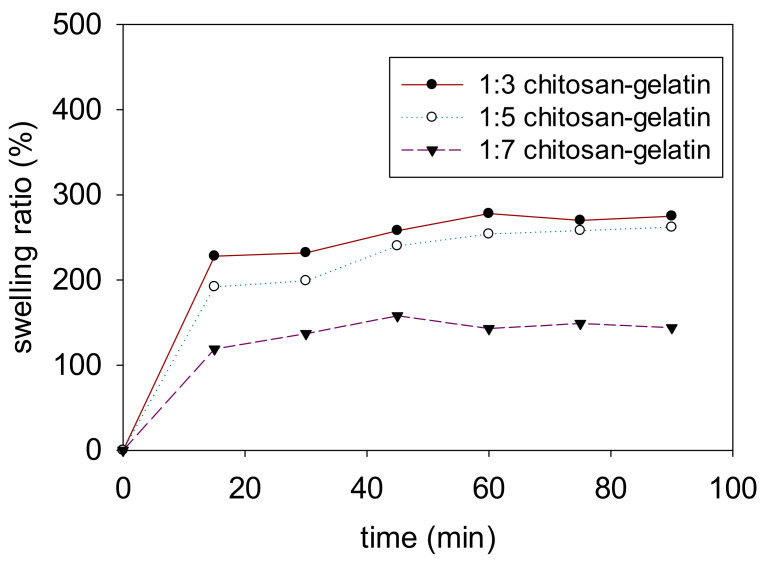
Swelling ratio percentage of the membrane with different composition ratios in pure water.

One of the important components in the separation process is the swelling behavior. The water-uptake capability of each sample was tested, and the membrane was tested in pure water, as shown in Figure 8. The swelling test was performed to determine the optimum composition ratio of chitosan and gelatin to investigate structure of the membrane stability t as a functionalization on a significant application as membrane filtrations. The absorption of water during the swelling test resulted in some changes in the membrane structure and size due to the hydrophilic function of the three-dimensional network porous structure. A higher composition of gelatin provides higher stability to the membrane structure.

The swelling testing was performed with different composition ratios of chitosan–gelatin, 1:3, 1:5, and 1:7. The highest swelling ratio value was observed for the chitosan–gelatin 1:3 membrane foam at an average percentage swelling ratio of 220%, whereas, in the chitosan:gelatin composition ratio of (1:5), the swelling ratio value was approximately 200% in both the media. This phenomenon suggested that the increase in the gelatin content decreased the swelling ratio, which was attributed to the increasing degree of crosslinking due to the increased number of functional amino groups. The results showed that a stable membrane foam was obtained at the chitosan:gelatin composition ratio of 1:7 in the crosslinked membrane with an average swelling ratio of approximately 121% of swelling in pure water. The stability of the membrane with the chitosan:gelatin composition ratio of 1:7 was strongly affected due to the higher gelatin content, which resulted in a higher degree of crosslinking. The use of crosslinkers in gelatin is an effective method to obtain stable covalent bonds among its polypeptides. Consequently, an improvement in the water resistance from rapid destruction in aqueous media allows the resulting membrane to be used many times in the water medium and this indicates that an increase in mechanical properties [39,40,41].

Strengthening the strength and durability of the material in the water medium is one of the important functional properties of bio-based materials in the separation process. Incorporating the 1:7 chitosan:gelatin crosslink with genipin affected the membrane swelling, with a moderate swelling ratio at the lowest percentage compared to the other composition ratio in pure water shows slightly higher in swelling ratio. The swelling ratio of the chitosan–gelatin and genipin membranes decreased with the increasing gelatin content, due to the higher functional group that resulted from the higher gelatin content that was treated with genipin to enhance the network entanglement chain with a better stability membrane and had a compact porous structure of membrane itself because the higher crosslinking density reduced the ability to absorb water. The swelling ratio decreased significantly with the increasing degree of crosslinking because of the increased crosslinking of the amino group in chitosan and gelatin (1:7) to form a denser microstructure [42]. The significant improvement in the stability of the membrane, which was mainly due to the strengthened intermolecular interactions between chitosan and gelatin, may constrain the interpenetrating polymer networks. This interlocked structure in the crosslinked networks enables one polymer chain to permeate into another polymeric network at molecular level with or without chemical bonds [24], thus preventing the water molecules from destroying the bio-based membrane [15].

### 3.8. Membrane Testing

**Figure 9:**
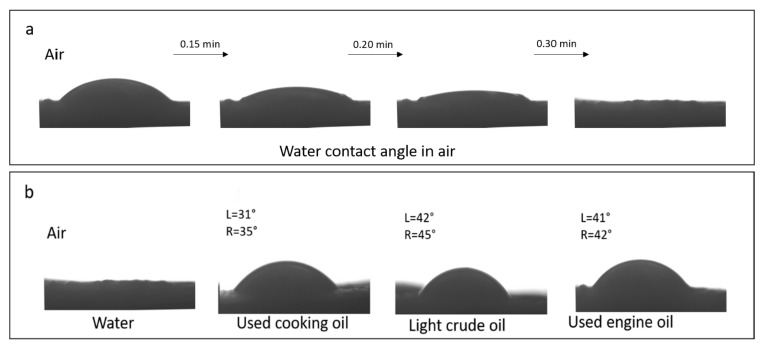
Modified (1:7) chitosan–gelatin membrane wetting behavior: (**a**) water wettability and (**b**) oil wettability in air.

The oil–water separation performance was analyzed to determine water permeability and oil resistance of the biopolymer membrane. The (1:7) chitosan–gelatin membrane with good water absorption, which was employed in this study, is a promising candidate for application in oil–water separation. Figure 9a shows the wettability test of the sample with water; Figure 9b shows the membrane testing results with different types of oil with water as a reference. Table 2 presents the average viscosity of each oil used in the study. The water contact angle was used to measure the wettability of the gelatin–chitosan 1:7 membrane foam in air with a 5 μL water droplet. As shown in Figure 9a, when the water droplet comes in contact the membrane foam surface, it takes a certain amount of time to spread completely on the surface, and it permeates into the sponge within 30 min. Therefore, the water contact angle is 0°. The hydrophilicity of the membrane foam produced is due to the strong intra- and intermolecular hydrogen bonding. The strong affinity of hydrogen bonding toward the water molecules and the external surface of the membrane results in uniform spreading and absorption of the membrane foam. However, when water was replaced with various oil droplets to study the wetting behavior, the spreading and adsorption differed. For each sample of oil used in application testing, a certain amount of oil (5 μL), the results show that oil droplets remain at a certain point on the membrane surface after 30 min. The results indicate that the membrane surface is oleophobic in air, with varying oil contact-angle values for used cooking oil (L = 31°, R = 35°), light crude oil (L = 42°, R = 45°) and used engine oil (L = 41°, R = 42°). All the oil contact angles on the membrane foam confirmed the oleophobic properties and indicated a stable membrane foam in a wide range of oil viscosities. The differences in the contact angle were a result of the roughness of the real surface of the membrane produced. The roughness resulted in an enlarged surface area and effected the wettability properties of the membrane. The contact angle was smaller on a rough material so the material was, therefore, even more wettable. This observation indicated the potential of the membrane for application in oil–water separation. In addition, oil droplets trapped at the membrane surface under water were observed in the membrane foam. This indicated that the chitosan–gelatin membrane foam was oleophobic under water. These wettability behaviors for different oils indicated the affinity of the membrane surface toward selective absorption and permeation of water.

**Figure 10:**
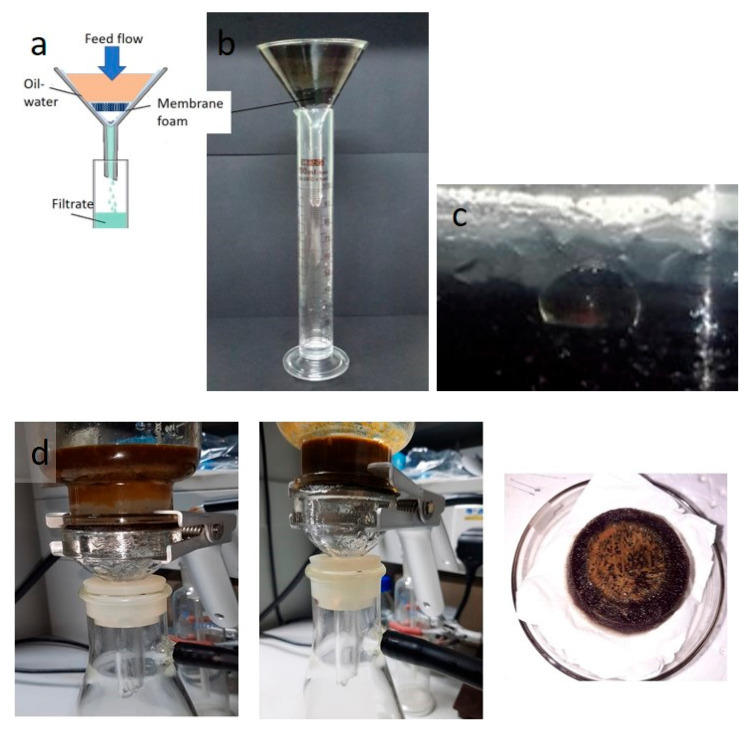
(**a**) Oil–water separation process using a funnel. (**b**) Membrane used to filter light crude oil–water. (**c**) Oil droplets in water at the membrane surface. (**d**) Used engine oil–water mixture before and after the separation; photograph of the membrane after the separation.

A small lab-scale sample was used to test the membrane form for separation performance. Figure 10a shows a mixture of water and oil poured into a glass funnel with the membrane placed in the neck of the funnel. Water selectively permeated through the (1:7) gelatin–chitosan membrane foam solely due to gravity, whereas the oil was repelled and retained in the upper tube. The oil–water separation process is shown in Figure 10b. The oil droplet rippled, coalesced, and was retained above the membrane foam surface owing to the oleophilic nature of the membrane; however, the water permeated through the membrane, owing to its hydrophilic nature. The separation depends on the fluid density differences in addition to the porosity and wettability of the separator.

**Figure 11:**
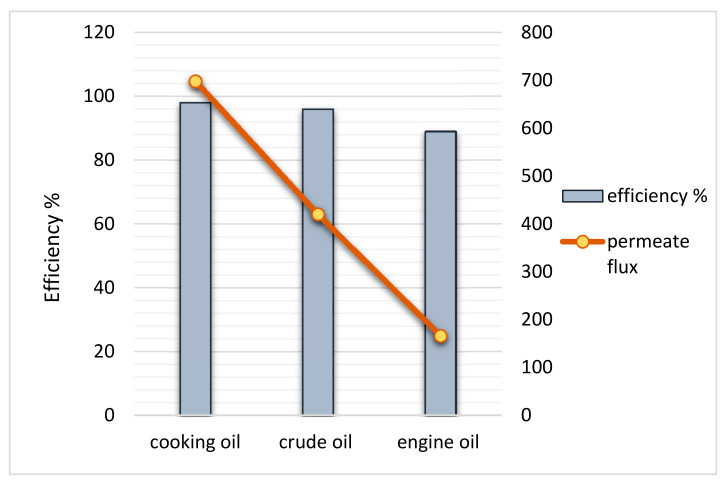
Percentage efficiency and flux (L∙m^−2^∙h^−1^) trends for the modified chitosan–gelatin membrane with used cooking oil, light crude oil, and used engine oil with water mixture.

The results of membrane testing to determine the membrane performance in the oil–water separation process revealed distinct performance with used cooking oil, light crude oil, and used engine oil–water mixture in a 1:2 volume ratio. Figure 11 shows the permeation flux and percentage efficiency of the separation process of various mixtures using the 1:7 chitosan–gelatin membrane foam. The separation of the oil–water phases was conducted under gravity to ensure that the water flows across the membrane, which was dependent on several membrane parameters such as porosity, pore size, thickness, and hydrophilicity [38,43]. The time taken for complete separation was evaluated. As mentioned earlier, the membrane was tested under different oil viscosities.

As shown in Figure 11, the initial flux results for application testing of mixture used cooking oil–water at a rate of 698 L∙m^−2^∙h^−1^, the mixture of light crude oil–water resulted in a rate of 420 L∙m^−2^∙h^−1^ fluxes yield. Meanwhile, for the used engine oil separation process, it is clear that the lowest flux yield was at 166 L∙m^−2^∙h^−1^. The separation pattern of the used cooking oil was much faster compared to light crude oil, even though the viscosity of the used cooking oil was slightly higher. This is because used cooking oil has highly concentrated oil and fat; therefore, water contaminated with this oil can be separated easily as the oil remains separated from the water [39]. The separation efficiency depends on the natural state of the oil and the feed viscosity during the separation process. However, the use of a light crude oil mixture for separation has a tendency to reduce the separation rate as the light oil due to the mixture of oil water and light crude oil turning into the emulsion in a significantly low viscosity was reflected by its surface tension and dispersed phases in water. Since water is the rich phase in the mixture, the light oil is significantly influenced by the hydrogen bonds and hydrodynamic forces [44]. The highly viscous feed increases the total resistance in the separation process. The lowest flux yield is obtained from the used engine oil–water mixture. The highly viscous used engine oil may contain toxic chemicals that can adhere to everything and cause contamination; therefore, it considered as the root cause of pollution. The separation becomes difficult because the engine oil is very viscous, and the contaminated water contains high oil content. Water contamination with the used engine oil can easily cause membrane fouling during the separation process, which tends to decrease the flux and efficiency of separation. Therefore, for a highly viscous oil–water contamination separation process, the membrane undergoes a cleaning process for the reusability of the membrane due to its hydrophilic properties by washing the membrane with water and soap to remove oil on the membrane surface. The hydrophilic membranes experienced a lower decrease in the overall flux as compared to the hydrophobic membranes clogging the separator with oil because of the high concentration of the oil, which indicates a higher resistance to oil–water filtration [45,46]. Oil adhering to the surface reduce the performance and reusability of the membrane. Utilizing a bio-membrane is one of the effective methods to improve the fouling behavior of the membrane to overcome the material’s resistance to fouling due to this membrane can be cleaned to remove material that causes it to clog by cleaning the surface removing oil contaminations. To clean the grease/oily membrane, especially when contaminated with high-viscosity used engine oil (Figure 10d), only soap and water are required. Squirt a tiny drop of liquid soap on the membrane and gently wipe the surface until the oil disappears; rinse with water. The cleaned membrane can be used several times, without causing any significant decrease in separation efficiency. The membrane must also be cleaned before disposal, if disposal as solid waste is desired.

## 4. Conclusions

In conclusion, bio-based chitosan–gelatin membranes were successfully fabricated with a stable structure by modifying them with the crosslinker genipin. A modified bio-based membrane foam was successfully fabricated to support the structure scaffold network for application in oil–water separation. This foam membrane was found to be a sustainable alternative for the synthetic membrane. The structural modifications by introducing the crosslinking genipin in the porous structure of the membrane were studied by FESEM micrographs. Dynamic-time-sweep and dynamic-train-sweep tests showed that the gelation time and strength of the hydrogel could be modulated by the content of genipin. With the increase in the genipin content, the gelation time decreased, and the strength of the hydrogel increased. The gelatin–chitosan membrane foam with a microporous structure exhibited a flux of separation efficiency greater than 98%. The three-dimensional scaffold network of the hydrophilic membrane foam exhibited excellent separation properties because of its high porosity, and it could be easily customized for the application in the oil–water separation process. The mechanical and rheological properties of the membrane were enhanced and had an optimum chitosan:gelatin composition ratio of 1:7. It was also observed that bio-based membrane features, such as sustainability, reduce the after-process serious environmental problems produced by the consumption of materials that are difficult to dispose and cause new pollution. This suggested a higher crosslinker content within the matrix, owing to the more functional groups with the increasing gelatin content. These results indicate that chitosan–gelatin and genipin are compatible and reflect the strong interaction between the matrix and the crosslinker. The porosity of the chitosan–gelatin membrane was further enhanced by increasing the chitosan–gelatin composition ratio, which can be utilized in the separation process with different oil viscosities.

## Figures and Tables

**Table 1:** Degradation temperature and mass residual percentage of the modified membrane foam at chitosan:gelatin composition ratios of 1:3, 1:5, and 1:7 during the TGA and DSC analyses.

Sample Ratio	TGA Analysis		DSC Analysis
Chitosan:Gelatin	T_d_ (°C)	Residual Mass (%)	T_d_ (°C)
1:03	319.3	17.6	67.95
1:05	329.1	19.91	79.28
1:07	331.5	17.96	87.3

**Table 2:** Average values of the viscosities of oils.

Type	Viscosity (cP)
Used cooking oil	107.7
Light crude oil	28.4
Used engine oil	135.0

## Data Availability

Not applicable.

## References

[1] Wang G., He Y., Zhang L., Yu Q., Peng S., Wu X., Ren T., Zeng Z., Xue Q. (2015). A cellulose sponge with robust superhydrophilicity and under-water superoleophobicity for highly effective oil/water separation. Green Chem..

[2] White I.C., Molly F.C. (2003). Factors that determine the cost of oil spills. Int. Oil spill Conf..

[3] Obotey E.E., Rathilal S. (2020). Membrane technologies in wastewater treatment: A review. Membranes.

[4] Prendergast D.P., Gschwend P.M. (2014). Assessing the performance and cost of oil spill remediation technologies. J. Clean. Prod..

[5] Zioui D., Salazar H., Aoudjit L., Martins P.M., Lanceros-Méndez S. (2020). Polymer-Based Membranes for Oily Wastewater Remediation. Polymers.

[6] Ahmad N.A., Goh P.S., Abdul Karim Z., Ismail A.F. (2018). Thin film composite membrane for oily waste water treatment: Recent advances and challenges. Membranes.

[7] Singh R., Hankins N. (2016). Emerging Membrane Technology for Sustainable Water Treatment.

[8] Arikibe J.E., Lata R., Kuboyama K., Ougizawa T., Rohindra D. (2019). pH-responsive studies of bacterial cellulose /chitosan hydrogels crosslinked with genipin: Swelling and drug release behaviour. ChemistrySelect.

[9] El-hefian E.A., Yahaya A.H. (2010). Rheological study of chitosan and its blends: An overview. Maejo Int. J. Sci. Technol..

[10] Yien L., Zin N.M., Sarwar A., Katas H. (2012). Antifungal activity of chitosan nanoparticles and correlation with their physical properties. Int. J. Biomater..

[11] Haug I.J., Draget K.I. (2009). Gelatin. Handbook of Hydrocolloids.

[12] Zhang Y., Wang Q.-S., Yan K., Qi Y., Wang G.-F., Cui Y.-L. (2016). Preparation, characterization, and evaluation of genipin crosslinked chitosan/gelatin three-dimensional scaffolds for liver tissue engineering applications. J. Biomed. Mater. Res. A.

[13] Tseng H.J., Tsou T.L., Wang H.J., Hsu S.H. (2013). Characterization of chitosan–gelatin scaffolds for dermal tissue engineering. J. Tissue Eng. Regen. Med..

[14] Kil’deevaa N.R., Kasatkina M.A., Mikhailov S.N. (2017). Peculiarities of obtaining biocompatible films based on chitosan cross linked by genipin. Polym. Sci. Ser. D.

[15] Zhaoxuan F., Karin O., Minna H. (2018). Tunable chitosan hydrogels for adsorption: Property control by biobased modifiers. Carbohydr. Polym..

[16] Wan Ishak W.H., Ahmad I., Ramli S., Mohd Amin M.C.I. (2018). Gamma Irradiation-Assisted Synthesis of Cellulose Nanocrystal-Reinforced Gelatin Hydrogels. Nanomaterials.

[17] Juthamas R., Ratthapol R., Hathairat J., Sorada K., Siriporn D. (2010). Influences of physical and chemical crosslinking techniques on electrospun type A and B gelatin fiber mats. Int. J. Biol. Macromol..

[18] Yuanyuan Z., Zhongtao S. (2017). Effects of gelatin-polyphenol and gelatin–genipin cross-linking on the structure of gelatin hydrogels. Int. J. Food Prop..

[19] Da Silva R.S.G., Pinto L.A.A. (2012). Physical crosslinkers: Alternatives to improve the mechanical properties of fish gelatin. Food Eng. Rev..

[20] Arif M.M.A., Fauzi M.B., Nordin A., Hiraoka Y., Tabata Y., Yunus M.H.M. (2020). Fabrication of Bio-Based Gelatin Sponge for Potential Use as A Functional Acellular Skin Substitute. Polymers.

[21] Pozzo L.D.Y., da Conceição T.F., Spinelli A., Scharnagl N., Pires A.T.N. (2018). Chitosan coatings crosslinked with genipin for corrosion protection of AZ31 magnesium alloy sheets. Carbohydr. Polym..

[22] Dimida S., Barca A., Cancelli N., De Benedictis V., Raucci M.G., Demitri C. (2017). Effects of genipin concentration on cross-linked chitosan scaffolds for bone tissue engineering: Structural characterization and evidence of biocompatibility features. Int. J. Polym. Sci..

[23] Moura M.J., Martins S.P., Duarte B.P. (2015). Production of chitosan microparticles cross-linked with genipin-identification of factors influencing size and shape properties. Biochem. Eng. J..

[24] Cui L., Xiong Z., Guo Y., Liu Y., Zhao J., Zhang C., Zhu P. (2015). Fabrication of interpenetrating polymer network chitosan/gelatin porous materials and study on dye adsorption properties. Carbohydr. Polym..

[25] Butler M., Ng Y.-F., Pudney P.D.A. (2003). Mechanism and kinetics of crosslinking reaction between biopolymers containing primary amine groups and genipin. J. Polym. Sci. Polym. Chem..

[26] Wang Q., Jiang J., Xiong Y.L. (2019). Genipin-aided protein cross-linking to modify structural and rheological properties of emulsion-filled hempseed protein hydrogels. J. Agric. Food Chem..

[27] Poursamar S.A., Lehner A.N., Azami M., Ebrahimi-Barough S., Samadikuchaksaraei A., Antunes A.P.M. (2016). The effects of crosslinkers on physical, mechanical, and cytotoxic properties of gelatin sponge prepared via in-situ gas foaming method as a tissue engineering scaffold. Mater. Sci. Eng. C.

[28] Noranizan I.A., Ahmad I. (2012). Effects of fiber loading and compatibilizer on rheological, mechanical and morphology behaviors. Open J. Polym. Chem..

[29] Ortiz-Zarama M.A., Jim’enez-Aparicio A.R., Lourenço R.V., Amaral-Sobral P.J., Solorza-Feria J. (2016). Rheological characterization of solutions of gelatin with bentonite and tannic acid. Rev. Mex. Ing. Quim..

[30] Moura M.J., Figueiredo M.M., Gil M.H. (2007). Rheological study of genipin cross-linked chitosan hydrogels. Biomacromolecules.

[31] Song Y., Nagai N., Saijo S., Kaji H., Nishizawa M., Abe T. (2018). In situ formation of injectable chitosan–gelatin hydrogels through double crosslinking for sustained intraocular drug delivery. Mater. Sci. Eng. C.

[32] Delmar K., Bianco-Peled H. (2015). The dramatic effect of small pH changes on the properties of chitosan hydrogels crosslinked with genipin. Carbohydr. Polym..

[33] Kota A.K., Kwon G., Choi W., Mabry J.M., Tuteja A. (2012). Hygro-responsive membranes for effective oil–water separation. Nat. Commun..

[34] Penga C., Zhaoa S.-Q., Zhanga J., Huanga G.-Y., Chena L.-Y., Zhao F.-Y. (2014). Chemical composition, antimicrobial property and microencapsulation of mustard (Sinapis alba) seed essential oil by complex coacervation. Food Chem..

[35] Cui L., Jia J.F., Guo Y., Liu Y., Zhu P. (2014). Preparation and characterization of IPN hydrogels composed of chitosan and gelatin cross-linked by genipin. Carbohydr. Polym..

[36] Shok Yin O., Ahmad I., Mohd Amin M.C.I. (2015). Effect of Cellulose Nanocrystals Content and pH on Swelling Behaviour of Gelatin Based Hydrogel. Sains Malays..

[37] Samsalee N., Sothornvit R. (2017). Modification and characterization of porcine plasma protein with natural agents as potential crosslinkers. Int. J. Food Sci. Technol..

[38] Miles C.A., Avery N.C., Rodin V.V., Bailey A.J. (2005). The increase in denaturation temperature following cross-linking of collagen is caused by dehydration of the fibres. J. Mol. Biol..

[39] Zhang X., Do M.D., Casey P., Sulistio A. (2010). Chemical cross-linking gelatin with natural phenolic compounds as studied by high-resolution NMR spectroscopy. Biomacromolecules.

[40] Alizadeh M., Abbasi F., Khoshfetrat A.B., Ghaleh H. (2013). Microstructure and characteristic properties of gelatin/chitosan scaffold prepared by a combined freeze-drying/leaching method. Mater. Sci. Eng. C.

[41] Tan X., Rodrigue D. (2019). A review on porous polymeric membrane preparation. Part I: Production techniques with polysulfone and poly (vinylidene fluoride). Polymers.

[42] Khayeta M., Menguala J.I., Matsuura T. (2005). Porous hydrophobic/hydrophilic composite membranes. Application in desalination using direct contact membrane distillation. J. Membr. Sci..

[43] Guo W., Zhu Y., Han Y., Wei Y., Luo B. (2017). Separation mechanism of fatty acids from waste cooking oil and its flotation performance in iron ore desiliconization. Minerals.

[44] Ariffina T.S.T., Yahyaa E., Husin H. (2016). The rheology of light crude oil and water-in-oil-emulsion. Procedia Eng..

[45] Bolto B., Zhang J., Wu X., Xie Z. (2020). A review on current development of membranes for oil removal from waste waters. Membranes.

[46] Elshorafa R., Saththasivam J., Liu Z., Ahzi S. (2020). Efficient oil/saltwater separation using a highly permeable and fouling-resistant all-inorganic nanocomposite membrane. Environ. Sci. Pollut. Res..

